# Continuous positive airway pressure titration in infants with severe upper airway obstruction or bronchopulmonary dysplasia

**DOI:** 10.1186/cc12846

**Published:** 2013-07-26

**Authors:** Sonia Khirani, Adriana Ramirez, Sabrina Aloui, Nicolas Leboulanger, Arnaud Picard, Brigitte Fauroux

**Affiliations:** 1 S2A Santé, Ivry sur Seine, France; 2 AP-HP, Hôpital Armand Trousseau, Pediatric Pulmonary Department, Paris, France; 3 ADEP Assistance, Suresnes, France; 4 AP-HP, Hôpital Armand Trousseau, Pediatric Otorhinolaryngology and Head and Neck Surgery Department, Paris, France; 5 Université Pierre et Marie Curie-Paris 6, Paris, France; 6 INSERM U 955, Créteil, France; 7 AP-HP, Hôpital Armand Trousseau, Pediatric Plastic Surgery and Maxillofacial Department, Paris, France

**Keywords:** Airway obstruction, Continuous positive airway pressure, Oesophageal pressure, Expiratory abdominal activity, Infant

## Abstract

**Abstracta:**

**Introduction**

Noninvasive continuous positive airway pressure (CPAP) is recognized as an effective treatment for severe airway obstruction in young children. The aim of the present study was to compare a clinical setting with a physiological setting of noninvasive CPAP in infants with nocturnal alveolar hypoventilation due to severe upper airway obstruction (UAO) or bronchopulmonary dysplasia (BPD).

**Methods**

The breathing pattern and respiratory muscle output of all consecutive infants due to start CPAP in our noninvasive ventilation unit were retrospectively analysed. CPAP set on clinical noninvasive parameters (clinical CPAP) was compared to CPAP set on the normalization or the maximal reduction of the oesophageal pressure (Poes) and transdiaphragmatic pressure (Pdi) swings (physiological CPAP). Expiratory gastric pressure (Pgas) swing was measured.

**Results**

The data of 12 infants (mean age 10 ± 8 mo) with UAO (*n *= 7) or BPD (*n *= 5) were gathered. The mean clinical CPAP (8 ± 2 cmH_2_O) was associated with a significant decrease in Poes and Pdi swings. Indeed, Poes swing decreased from 31 ± 15 cmH_2_O during spontaneous breathing to 21 ± 10 cmH_2_O during CPAP (*P *< 0.05). The mean physiological CPAP level was 2 ± 2 cmH_2_O higher than the mean clinical CPAP level and was associated with a significantly greater improvement in all indices of respiratory effort (Poes swing 11 ± 5 cm H_2_O; *P *< 0.05 compared to clinical CPAP). Expiratory abdominal activity was present during the clinical CPAP and decreased during physiological CPAP.

**Conclusions**

A physiological setting of noninvasive CPAP, based on the recording of Poes and Pgas, is superior to a clinical setting, based on clinical noninvasive parameters. Expiratory abdominal activity was present during spontaneous breathing and decreased in the physiological CPAP setting.

## Introduction

Noninvasive continuous positive airway pressure (CPAP) is recognized as an effective treatment for severe upper airway obstruction (UAO) in young children. Indeed, the maintenance of airway patency throughout the entire breathing cycle by means of CPAP has been shown to be associated with an unloading of the respiratory muscles, an improvement of breathing pattern and gas exchange [1-6].

In adults and adolescents with obstructive sleep apnoea (OSA), the titration of CPAP is based on the polysomnographic disappearance of apnoea, hypopnoea, respiratory effort-related arousal and snoring, as recommended by the American Academy of Sleep Medicine (AASM) [7,8]. The diseases responsible for airway obstruction in infants differ from those of older children and adults with the predominance of anatomical abnormalities of the upper airways such as laryngomalacia or tracheomalacia, Pierre Robin syndrome or other maxillofacial malformations [3,6].

Some other diseases involving the lower respiratory tract, such as chronic lung diseases of prematurity, also called *bronchopulmonary dysplasia *(BPD), are associated with lung diseases and predominantly peripheral airway obstruction, which may be severe and cause intrinsic positive end-expiratory pressure (PEEPi). Because of these differences in pathophysiology and the lack of guidelines for this age group, the titration of CPAP in infants is generally based on clinical parameters such as the disappearance of the stridor and retractions, the decrease in respiratory and heart rates and the normalization of gas exchange [2,3]. To facilitate the acclimatization of the infant with CPAP, the initial level is usually set at 4 cmH_2_O, followed by a gradual increase of the CPAP level until the best clinical efficacy and comfort are obtained.

Importantly, small changes in the CPAP level may have significant clinical consequences. Indeed, the minimal airway diameter is the most critical because, according to Poiseuille's law, the resistance increases with an exponent 4 of the radius. This has been observed in infants with acute viral bronchiolitis, in whom a 2 cmH_2_O change of CPAP level was associated with a significant change in the work of breathing and breathing pattern [9]. In such a homogeneous group of infants, the optimal CPAP level, determined by monitoring the oesophageal (Poes) and gastric pressure (Pgas), was 7 cmH_2_O for all the patients; however, it is not known if this level is also appropriate for infants with UAO and BPD, in whom CPAP could counteract the PEEPi.

The aim of the present study was to compare two settings of CPAP: a first setting based on noninvasive clinical parameters and a second setting based on the invasive recording of Poes and Pgas in a group of infants with severe UAO or BPD.

## Materials and methods

### Patients

All the data of consecutive infants less than 24 months old with severe UAO or BPD evaluated for noninvasive CPAP in a noninvasive ventilation unit of a paediatric university hospital were retrospectively analysed. All the patients with UAO had a laryngotracheal endoscopy under general anaesthesia. The UAO persisted in all the UAO patients despite endoscopic resection of the aryepiglottic folds and antireflux treatment using proton pump inhibitors. All the UAO and BPD patients were hypoxaemic (pulse oximetry (SpO_2_) less than 90% for more than 10 consecutive min and/or more than 10% of sleep time) and hypercapnic (transcutaneous carbon dioxide pressure (PtcCO_2_) above 50 mmHg for more than 10 consecutive min and/or more than 10% of sleep time) during an overnight sleep study [6]. Exclusion criteria were severe mental retardation and important midfacial deformity precluding the tolerance of a nasal mask. The study was approved by the Institutional Review Board of the French learned society for respiratory medicine, Société de Pneumologie de Langue Française (CEPRO 2012-031), and all the parents gave informed consent for the CPAP evaluation of their children.

### CPAP equipment

Because of the lack of adequate nasal interfaces for infants, all the patients were equipped with custom-made nasal masks manufactured by the prosthetists of the maxillofacial department of our hospital [10,11]. The CPAP devices used for the study were the Synchrony or Harmony (Philips Respironics, Murrysville, PA, USA), S9 or VPAP IV (ResMed, Bella Vista, Australia) and ICON (Fisher & Paykel Healthcare, Auckland, NZ) with an integrated humidification system. A commercial simple line circuit, recommended by the manufacturer, was connected to the nasal mask via an exhalation valve (Plateau Exhalation Valve; Philips Respironics). Oxygen therapy was not used during the study.

### Measurements

Airway pressure (Paw) was measured with a differential pressure transducer (MP45 Low Pressure Transducer model ± 100 cmH_2_O; Validyne Engineering Corp, Northridge, CA, USA) on the Plateau Exhalation Valve during CPAP treatment. We were not able to measure airflow during spontaneous breathing, because the infants did not tolerate the mask alone with the pneumotachograph due to the increase in dead space and during CPAP because of the constant airflow.

Poes and Pgas were measured using a 2.1-mm external diameter catheter mounted pressure transducer system with two integrated pressure transducers (Gaeltec, Dunvegan, Isle of Skye, UK) inserted pernasally after careful local anaesthesia (lidocaine 2%; AstraZeneca, Rueil-Malmaison, France) [12,13]. After calibration, the catheter was advanced gently until the distal tip was in the stomach and the proximal pressure transducer was in the middle portion of the oesophagus. Appropriate placement of the Poes transducer was assessed using the occlusion technique [14]. Adequate placement of the Pgas transducer was ascertained by gentle manual pressure on the patient's abdomen to observe fluctuations in Pgas, which should be absent on the Poes trace. Transdiaphragmatic pressure (Pdi) was obtained by online subtracting of the Poes signal from the Pgas signal. All the signals were digitized at 128 Hz and sampled for analysis using an analogical/numeric acquisition system (MP100 System; *BIOPAC *Systems, Inc, Goleta, CA, USA), run on a PC computer (Elonex, Gennevilliers, France) and displayed using Acq*Knowledge *software (*BIOPAC *Systems, Inc). SpO_2_, heart rate and respiratory rate (*f*R) were recorded.

### Protocol

No sedation was administered. The procedure was performed with the presence of at least one parent (generally the mother) and started with the placement of the oesogastric catheter at the time of a natural daytime nap. After 10 min of quiet breathing (ideally sleep), a 2-min baseline recording (spontaneous breathing) was taken. Next, the oesogastric recording was blinded to the investigator, and CPAP was started with an initial CPAP level at 4 cmH_2_O followed by a 1 cmH_2_O increase every 5 min until maximal clinical improvement. The clinical CPAP level considered optimal was the level associated with the disappearance of the stridor or retractions, a SpO_2 _greater than 95% on room air, the normalization or greatest decrease in heart rate and *f*_R_ and the best comfort as assessed by the infant's remaining or falling asleep. After 10 min of quiet breathing at this CPAP level, a 2-min recording (clinical CPAP) was performed. Thereafter the CPAP level was decreased to the baseline value of 4 cmH_2_O for 5 min. After this period, the oesogastric recording was revealed to the investigator. The CPAP level was then adjusted on the normalization or the maximal decrease in the Poes swing, independently of the clinical parameters. After 10 min of quiet breathing at this CPAP level, a 2-min recording (physiological CPAP) was performed. Next, the CPAP level was increased by 1 cmH_2_O. A 2-min recording (physiological CPAP+1) was performed after 10 min of quiet breathing. The long-term CPAP use and clinical outcome were assessed in all patients.

### Data analysis

*f*_R_ and inspiratory time/total respiratory cycle time (T_I_/T_TOT_) ratio were estimated from the Poes trace. Poes and Pdi swings and the oesophageal pressure time product (PTPoes) and diaphragmatic pressure-time product (PTPdi) per breath and per minute were measured and calculated as previously described [12,15-17]. Normally, PTPoes is measured as the area subtended by Poes and the chest wall static recoil pressure-time curve over the T_I_, taking into account the PEEPi, as described by Sassoon *et al*. [15]. As it was impossible to measure airflow in these infants, owing to the increase in dead space when a face mask was added or during CPAP because of the continuous positive airflow, we could not measure the real T_I_. For the calculation of the PTPoes/breath, the onset of inspiration was thus defined as the deflection on the Poes trace and the end of the inspiration as the peak of Pdi [18]. The PTPdi/breath was obtained by measuring the area under the Pdi signal from the onset of its positive deflection to its return to baseline. Both PTPoes and PTPdi were also expressed per minute by multiplying the pressure-time products per breath by the *f*_R_ (PTPoes/min and PTPdi/min) [15]. The Pgas swing during expiration was measured as a marker of expiratory abdominal activity. On the Pgas trace, we measured the decrease from the maximal end-expiratory level to the minimal value [19]. The presence of a positive Pgas swing during expiration was considered abnormal [20].

### Statistical analysis

Data are given as means ± SD. Comparisons between the baseline and different CPAP conditions were made using (1) one-way or one-factor repeated-measures analysis of variance (ANOVA) if data were normally distributed or (2) Friedman repeated-measures ANOVA on ranks if data were not normally distributed. When a significant difference was observed, pairwise comparisons were performed using the Holm-Sidak test. The CPAP levels were compared using a *t*-test if data were normally distributed or a Mann-Whitney *U *test if data were not normally distributed. A *P *value less than 0.05 was considered statistically significant.

## Results

**Figure 1:**
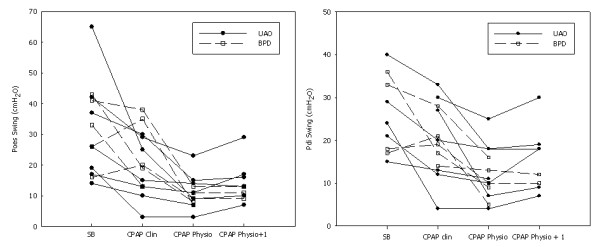
**Evolution of the oesophageal pressure (Poes) swing and the transdiaphragmatic pressure swing (Pdi) during spontaneous breathing (SB), a clinical setting of noninvasive continuous positive airway pressure (CPAP Clin), a physiological setting of noninvasive continuous positive airway pressure (CPAP Physio) and a physiological setting of noninvasive CPAP+1 cmH2O (CPAP Physio + 1)**. BPD: bronchopulmonary dysplasia, UAO: upper airway obstruction.

**Figure 2:**
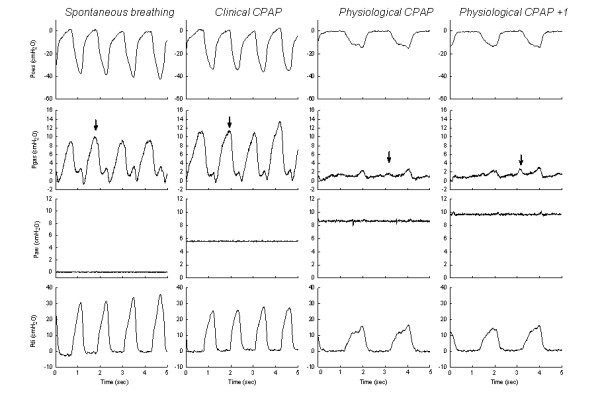
**Tracings of patient 9 showing the increases in oesophageal pressure (Poes) swing and transdiaphragmatic pressure (Pdi) swing during spontaneous breathing, together with an approximately 10 cmH_2_O expiratory gastric pressure (Pgas) swing (arrow)**. During a clinical setting of noninvasive continuous positive airway pressure (CPAP Clin) (that is, 6 cmH_2_O), a moderate decrease in the Poes and Pdi swing is observed with the persistence of an approximately 10 cmH_2_O expiratory gastric pressure (Pgas) swing (arrow). During a physiological setting of CPAP (CPAP Phys), note the important reduction in the Poes and Pdi swings and in the breathing rate with the disappearance of the expiratory Pgas swing (arrow). When the physiological CPAP setting is increased by 1 cmH_2_O, a moderate increase in expiratory Pgas swing is observed.

Twelve infants (mean post natal age 10 ± 8 mo, mean weight 6.6 ± 2.3 kg) were enrolled in the study (Table 1). Seven infants had severe UAO, and five infants had BPD. Spontaneous breathing pattern showed high values of *f*_R_, T_I_/T_TOT_ ratio and respiratory effort, as shown by the increase in the Poes and Pgas swings, PTPoes/breath and PTPdi/breath, and PTPoes/min and PTPdi/min (Tables 2 and 3 and Figures 1 and 2). The mean *f*_R_ tended to be slightly higher for the BPD patients (54 ± 6 breaths/min) than in the UAO patients (51 ± 12 breaths/min; *P *= 0.612) and their T_I_/T_TOT_ ratio was significantly lower (0.4 ± 0.1 vs 0.6 ± 0.1 for the UAO patients; *P *= 0.003). All the markers of respiratory effort, except the Pgas swing during expiration (7 ± 4 vs 1 ± 2 for the UAO patients; *P *= 0.03), were statistically comparable.

**Table 1:** Description of the patients.

Patients	Gender	Age (mo)	Weight (kg)	Underlying disease
1	Girl	2	3.6	Pierre Robin syndrome
2	Boy	2	5.0	Prader-Willi syndrome
3	Girl	10	6.7	Obstructive sleep apnoea
4	Boy	2	4.5	Laryngomalacia and Down syndrome
5	Boy	3	5.1	Laryngomalacia
6	Boy	4	5.2	Laryngomalacia
7	Girl	13	9.0	Laryngomalacia
8	Boy	5	4.4	Bronchopulmonary dysplasia
9	Boy	14	8.3	Bronchopulmonary dysplasia
10	Girl	20	8.5	Bronchopulmonary dysplasia
11	Boy	22	9.0	Bronchopulmonary dysplasia
12	Boy	22	10.3	Bronchopulmonary dysplasia

**Figure 3:**
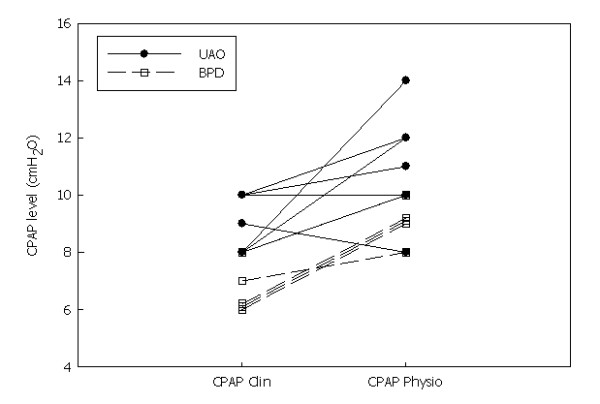
**Comparison of the noninvasive continuous positive airway pressure (CPAP) level during the clinical CPAP (CPAP Clin) setting and the physiological setting (CPAP Physio)**. BPD: bronchopulmonary dysplasia, UAO: upper airway obstruction.

The mean clinical CPAP level was 8 ± 2 cmH_2_O (range, 6 to 10 cmH_2_O) (Figure 3). This level was associated with a significant decrease in Poes and Pdi swings (Table 2). Of note, the mean clinical CPAP level was significantly higher for the UAO patients (9 ± 1 cmH_2_O) than for the BPD patients (7 ± 1 cmH_2_O) (*P *= 0.006) (Table 3).

**Table 2:** Breathing pattern, respiratory muscle output and continuous positive airway pressure level during spontaneous breathing at clinical and two physiological settings of continuous positive airway pressure in the total population.^a^

	Spontaneous breathing	Clinical CPAP	Physiological CPAP	Physiological CPAP+1 cmH_2_O
Respiratory parameters				
*f*_R _(breaths/min)	49 ± 14	44 ± 21	38 ± 16^b^	36 ± 18
T_I_/T_TOT_	0.5 ± 0.1	0.4 ± 0.1	0.4 ± 0.1^c^	0.4 ± 0.1
Respiratory muscle output				
Poes swing (cmH_2_O)	31 ± 15	21 ± 10^d^	11 ± 5^b^	14 ± 6
Pdi swing (cmH_2_O)	26 ± 8	20 ± 8^d^	12 ± 6^b^	15 ± 7
Pgas swing (cmH_2_O)	4 ± 2	3 ± 1	2 ± 1^c^	2 ± 1
Expiratory Pgas swing (cmH_2_O)	4 ± 4	3 ± 5	1 ± 1	2 ± 2
PTPoes/breath (cmH_2_O/s)	12 ± 6	9 ± 6	6 ± 5^b^	9 ± 9
PTPdi/breath (cmH_2_O/s)	11 ± 4	9 ± 6	6 ± 4^b^	8 ± 6
PTPoes/min (cmH_2_O/s/min)	571 ± 340	359 ± 227	183 ± 119^e^	244 ± 161
PTPdi/min (cmH_2_O/s/min)	452 ± 146	350 ± 164	201 ± 108^b^	239 ± 173
CPAP level (cmH_2_O)	NA	8 ± 2	10 ± 2	11 ± 2

^a^CPAP: continuous positive airway pressure, *f*_R_: respiratory rate, NA: not applicable, Poes: oesophageal pressure, Pdi: transdiaphragmatic pressure, Pgas: gastric pressure, PTP: pressure-time product, T_I_/T_TOT_: inspiratory time/total respiratory cycle time ratio. b*P *< 0.05 between physiological CPAP and spontaneous breathing or clinical CPAP. c*P *< 0.05 between spontaneous breathing and physiological CPAP. d*P *< 0.05 between spontaneous breathing and clinical CPAP. e*P *< 0.05 between physiological CPAP and spontaneous breathing, physiological CPAP or physiological CPAP+1 cmH_2_O.

**Table 3:** Breathing pattern, respiratory muscle output and continuous positive airway pressure level during spontaneous breathing at clinical and two physiological settings of continuous positive airway pressure for the upper airway obstruction and bronchopulmonary dysplasia patients.^a^

UAO (*n *= 7)	Spontaneous breathing	Clinical CPAP	Physiological CPAP	Physiological CPAP+1 cmH_2_O
Respiratory parameters				
*f*_R _(breaths/min)	46 ± 16^b^	36 ± 19	34 ± 17	32 ± 20
T_I_/T_TOT_	0.6 ± 0.1^b^	0.5 ± 0.1	0.4 ± 0.1	0.4 ± 0.1

Respiratory muscle output				
Poes swing (cmH_2_O)	30 ± 17^b^	18 ± 10	12 ± 6	16 ± 7
Pdi swing (cmH_2_O)	25 ± 9^b^	20 ± 10^c^	14 ± 7	17 ± 8
Pgas swing (cmH_2_O)	5 ± 2^b^	3 ± 1	3 ± 1	3 ± 1
Expiratory Pgas swing (cmH_2_O)	1 ± 2	1 ± 1	1 ± 1	2 ± 2
PTPoes/breath (cmH_2_O/s)	13 ± 7	10 ± 8	7 ± 6	11 ± 10
PTPdi/breath (cmH_2_O/s)	12 ± 5	11 ± 7	7 ± 5	10 ± 7
PTPoes/min (cmH_2_O/s/min)	599 ± 415^b^	291 ± 162	201 ± 150	271 ± 188
PTPdi/min (cmH_2_O/s/min)	459 ± 155^b^	332 ± 161^c^	212 ± 135	262 ± 193

CPAP level (cmH_2_O)	NA	9 ± 1	11 ± 2	12 ± 2

**BPD (*n *= 5)**	**Spontaneous Breathing**	**Clinical CPAP**	**Physiological CPAP**	**Physiological CPAP+1 cmH_2_O**

Respiratory parameters				
*f*_R _(breaths/min)	54 ± 6	56 ± 18	45 ± 13	46 ± 11
T_I_/T_TOT_	0.4 ± 0.0	0.4 ± 0.0	0.4 ± 0.1	0.4 ± 0.1

Respiratory muscle output				
Poes swing (cmH_2_O)	32 ± 11^d^	25 ± 11^c^	10 ± 2	11 ± 2
Pdi swing (cmH_2_O)	27 ± 9^d^	20 ± 5^c^	11 ± 4	11 ± 1
Pgas swing (cmH_2_O)	2 ± 1	2 ± 1	2 ± 1	2 ± 0
Expiratory Pgas swing (cmH_2_O)	7 ± 4	7 ± 6	1 ± 1	1 ± 1
PTPoes/breath (cmH_2_O/s)	10 ± 4^d^	9 ± 4^c^	4 ± 2	4 ± 2
PTPdi/breath (cmH_2_O/s)	9 ± 4	7 ± 3	5 ± 2	3 ± 0
PTPoes/min (cmH_2_O/s/min)	526 ± 201^d^	468 ± 290^c^	156 ± 43	181 ± 43
PTPdi/min (cmH_2_O/s/min)	442 ± 153	379 ± 183	183 ± 50	156 ± 0

CPAP level (cmH_2_O)	NA	7 ± 1	9 ± 1	10 ± 1

^a^BPD: bronchopulmonary dysplasia, CPAP: continuous positive airway pressure, *f*_R_: respiratory rate, NA: not applicable, Poes: oesophageal pressure, Pdi: transdiaphragmatic pressure, Pgas: gastric pressure, PTP: pressure time product, T_I_/T_TOT_: inspiratory time/total respiratory cycle time ratio, UAO: upper airway obstruction. b*P *< 0.05 between spontaneous breathing and clinical CPAP or physiological CPAP or physiological CPAP+1 cmH_2_O. c*P *< 0.05 between clinical CPAP and physiological CPAP. d*P *< 0.05 between spontaneous breathing and physiological CPAP or physiological CPAP+1 cmH_2_O.

The mean physiological CPAP level was 2 ± 2 cmH_2_O higher than the mean clinical CPAP level and was associated with a significantly greater improvement in *f*_R_, T_I_/T_TOT_ ratio and all the indices of respiratory effort as compared to spontaneous breathing (Table 2). Interestingly, the mean physiological CPAP level was not significantly different between the two groups (with a mean level of CPAP of 11 ± 2 cmH_2_O and 9 ± 1 cmH_2_O for the UAO and BPD patients, respectively; *P *= 0.12). The physiological CPAP+1 was associated with a nonsignificant increase in the Poes swing and the PTPoes/min, together with agitation and arousals in all the patients.

**Figure 4:**
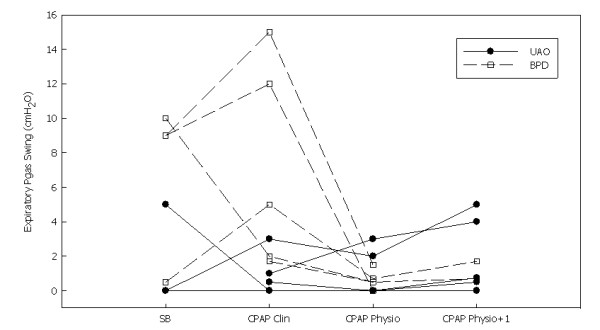
**Evolution of the gastric pressure (Pgas) swing during expiration during spontaneous breathing (SB), a clinical setting of noninvasive continuous positive airway pressure (CPAP Clin), a physiological setting of noninvasive continuous positive airway pressure (CPAP Physio) and a physiological setting of noninvasive CPAP+1 cmH_2_O) (CPAP Physio + 1)**. BPD: bronchopulmonary dysplasia, UAO: upper airway obstruction.

During spontaneous breathing, one of the five UAO patients and all four BPD patients had a Pgas swing during expiration (Pgas was not available for three patients due to technical reasons) (Figure 4). During the clinical CPAP setting, three of the seven UAO patients and all five BPD patients had a Pgas swing during expiration. During the physiological CPAP setting, this expiratory abdominal activity disappeared in one of the five BPD patients but persisted or increased in two UAO patients (Figure 4). During the physiological CPAP+1 setting, the expiratory abdominal activity increased in these two UAO patients, slightly reappeared in two other UAO patients and increased moderately in the two BPD patients (Pgas was not available in the other three BPD patients).

The physiological CPAP level was chosen for the patients. The clinical tolerance of this level was excellent, with all patients sleeping more than 6 h per night with CPAP and the normalization of nocturnal gas exchange, defined by a minimal SpO_2 _greater than 95% and a maximal PtcCO_2 _less than 50 mmHg. All the patients were successfully discharged to home on long-term nasal CPAP, and all were weaned from CPAP after a median period of 10 mo (range, 6 mo to 3 yr). CPAP was associated with an improvement in weight in all the patients. Three patients could be weaned from nasogastric nutritional support after 1 to 2 mo, and, in the other nine patients, CPAP was associated with an at least 1 SD weight gain after 1 to 3 mo. None of the patients required a tracheotomy, and no deaths occurred.

## Discussion

This study shows that a CPAP setting based on clinical noninvasive parameters is associated with an improvement in breathing pattern and respiratory effort in infants with severe UAO or BPD, but this improvement is significantly greater with an invasive physiological setting based on the monitoring of Poes and Pgas tracings.

We evaluated two groups of patients. The first group had severe UAO, in whom noninvasive CPAP has proven to be efficacious [3,6]. The second group comprised patients with severe BPD. We acknowledge that noninvasive CPAP is not a classic treatment for these infants, but the patients evaluated for CPAP in the present study represent a highly selected group of patients with extremely severe lung disease [21,22], as documented by the tremendous increase in work of breathing and alveolar hypoventilation. These patients are expected to have a combination of lower airway obstruction and parenchymal lung disease. The lower airway obstruction causes hyperinflation and PEEPi, which is effectively counteracted by CPAP, unloading the respiratory muscles and improving breathing pattern and gas exchange [23]. Despite an older age, the mean *f*_R_ tended to be higher and the mean T_I_/T_TOT_ ratio lower in the BPD patients than in the UAO patients, which may be explained by the predominantly lower airway obstruction in BPD patients. This may also explain the high prevalence of abnormal expiratory abdominal activity during spontaneous breathing in BPD patients.

The mean difference between the clinical CPAP and physiological CPAP levels was 2 ± 2 cmH_2_O. A 1 cmH_2_O increase in this physiological CPAP level was not tolerated by the infants with clinical arousal and an increase in expiratory abdominal effort, as assessed on the Pgas tracing. This narrow therapeutic CPAP range is similar to our findings in infants hospitalized for acute viral bronchiolitis [9]. These observations underline the sensitivity of these infants to small changes in the CPAP level.

Our findings regarding the expiratory Pgas recording are particularly interesting. Expiratory abdominal activity can be due to hyperinflation induced by CPAP overtitration, as shown by studies in healthy awake subjects in whom high levels of CPAP induced an expiratory abdominal activity [24-26]. In our group of patients, however, our hypothesis is that this expiratory abdominal activity was the consequence of persistent airway obstruction during spontaneous breathing and CPAP. Indeed, four of nine of our patients had expiratory abdominal activity during spontaneous breathing, which persisted during the clinical CPAP setting, as observed by Lofaso *et al*. [19]. Indeed, these authors monitored Poes and Pgas with airflow during nasal CPAP titration in 12 adult patients with OSA and observed that 6 patients presented expiratory abdominal activity during spontaneous breathing. This expiratory abdominal activity persisted at the CPAP level associated with the disappearance of apnoea and disappeared only when a higher CPAP level was reached [19]. The importance of this CPAP undertitration has recently been highlighted by Seo *et al*. [27]. These authors analysed the expiratory abdominal muscle activity by means of surface electrodes in 81 patients with OSA and periodic limb movements (PLMs) during CPAP titration [27]. PLMs persisted in 72 patients while the AASM-defined hypopnoea were eliminated [7]. These subjects still showed a pattern of flow limitation and the presence of cyclic alternative pattern during non-rapid eye movement sleep. The disappearance of flow limitation required a higher CPAP pressure and was associated with the disappearance of PLMs. When comparing the CPAP pressure necessary to eliminating AASM-defined hypopnoea and the pressure required to eliminating PLMs, Seo *et al*. found a mean difference of 1.47 ± 1.96 cmH_2_O. Forty-four patients needed 2 cmH_2_O or greater CPAP increase for the disappearance of flow limitation and PLM. The clinical relevance of this high-target CPAP titration seems important, as PLMs may be associated with evidence of electroencephalographic changes of arousal and with changes in the plethysmographic curve obtained from a finger oximeter, which indicates sympathetic stimulation [28]. Of note, the mean CPAP difference in the present study was similar to that observed in adult patients [27].

Our data underline the importance of expiratory abdominal activity for optimal titration of CPAP in infants, but an oesogastric pressure recording is not available in most centres. Surface electromyography of the abdominal muscles is probably a promising tool and worth being developed and validated for use in young infants. In the meantime, it has to be noted that the clinical CPAP level was associated with an improvement in breathing pattern and respiratory effort, as well as excellent clinical tolerance, with the majority of the patients falling asleep. A first recommendation may thus be to set CPAP at the level associated with the disappearance of the stridor, SpO_2 _greater than 95%, the greatest decrease in heart rate and *f*_R_, and the best comfort as assessed by the infant falling asleep. As the mean difference between the clinical CPAP level and the physiological CPAP level was about 2 cmH_2_O, we recommend increasing the clinical CPAP level by 1 to 2 cmH_2_O if this level remains associated with the disappearance of the stridor, a SpO_2 _greater than 95%, the greatest decrease in heart rate and *f*_R_ and the best comfort as assessed by the infant's remaining or falling asleep.

Our study has several limitations. We were unable to measure airflow and thus could not accurately report the mechanical inspiratory time during spontaneous breathing. Indeed, the increase in dead space following the application of the nasal mask resulted in an increase in respiratory effort and dyspnoea associated with a fall in SpO_2_. Also, the occurrence of a continuous high airflow during CPAP precluded the measurement of the patient's own airflow. We did not monitor gas exchange in all the infants during the study period. All the infants had severe hypoxaemia and hypercapnia during sleep, which was alleviated by nasal CPAP as previously reported by our group in a comparable group of patients [3,5,6]. Despite the fact that the infants fell asleep with the nasal CPAP, we did not perform an overnight recording. This is a limitation, as the most efficient pressure level may change with the sleep stage. Different CPAP devices were used. CPAP devices, as is true of other home ventilators, evolve very rapidly. As CPAP is the most simple ventilator mode, delivering only a constant positive pressure, the choice of the device is mainly a question of ergonometry, prescriber or patient preference. Indeed, we did not use more recent CPAP modes, such as bilevel, auto-adjusted and BiPAP Bi-Flex modes, which are not adapted for infants as we have shown previously [5]. We acknowledge that a small difference may be observed between the CPAP level set on the device and the Paw measured by the pressure transducer. In the present study, however, the CPAP level reported was the Paw and not the level given by the CPAP device. We did not perform a polysomnography and are thus unable to confirm the association between persistent respiratory effort and cortical arousal.

## Conclusions

This study shows that a physiological setting of noninvasive CPAP, based on the recording of Poes and Pgas, may be superior to a clinical setting based on clinical noninvasive parameters. Expiratory abdominal activity is worth detecting, underlining the importance of the development and validation of noninvasive, reliable and accurate markers of abdominal activity for an optimal setting of noninvasive CPAP in infants.

## Key messages

• In routine care, CPAP level should be adjusted on the disappearance of the stridor, retraction and/or dyspnoea, a pulse oximetry greater than 95%, the greatest decrease in heart and respiratory rates, and the best comfort as assessed by the infant's falling asleep.

• A setting of CPAP based on the recording of Poes and Pgas may be superior to a clinical setting.

• Expiratory abdominal activity may be helpful for an optimal titration of CPAP in infants.

## Abbreviations

AASM: American Academy of Sleep Medicine; BPD: bronchopulmonary dysplasia; CPAP: continuous positive airway pressure; *f*_R_: respiratory rate; OSA: obstructive sleep apnoea; Paw: airway pressure; Pdi: transdiaphragmatic pressure; PEEPi: intrinsic positive end-expiratory pressure; Pgas: gastric pressure; PLM: periodic limb movement; Poes: oesophageal pressure; PtcCO_2_: transcutaneous carbon dioxide pressure; PTPoes: oesophageal pressure-time product; PTPdi: diaphragmatic pressure time product; SpO_2_: pulse oximetry; T_I_/T_TOT_: inspiratory time/total respiratory cycle time; UAO: upper airway obstruction

## Competing interests

The authors declare that they have no competing interests.

## Authors' contributions

SK performed the analysis of tracings and the redaction of the manuscript. AR and SA helped with recording of the patients, analysis of the data and redaction of the manuscript. NL contributed to the recruitment of the patients, analysis of tracings and redaction of the manuscript. AP performed the nasal masks and contributed to the analysis of tracings and redaction of the manuscript. BF contributed to the study concept and design, recruitment of the patients, analysis of tracings and redaction of the manuscript. All authors read and approved the final manuscript.
